# Identification of molecular markers and candidate regions associated with grain number per spike in Pubing3228 using SLAF-BSA

**DOI:** 10.3389/fpls.2024.1361621

**Published:** 2024-03-05

**Authors:** Jiansheng Wang, Erwei Wang, Shiping Cheng, Aichu Ma

**Affiliations:** ^1^ College of Chemistry and Environment Engineering, Pingdingshan University, Pingdingshan, Henan, China; ^2^ Henan Key Laboratory of Germplasm Innovation and Utilization of Eco-economic Woody Plant, Pingdingshan, Henan, China; ^3^ Pingdingshan Academy of Agricultural Science, Pingdingshan, Henan, China

**Keywords:** bulk segregation analysis, grain number per spike, pubing3228, SNP, yield

## Abstract

Grain number per spike, a pivotal agronomic trait dictating wheat yield, lacks a comprehensive understanding of its underlying mechanism in Pubing3228, despite the identification of certain pertinent genes. Thus, our investigation sought to ascertain molecular markers and candidate regions associated with grain number per spike through a high-density genetic mapping approach that amalgamates site-specific amplified fragment sequencing (SLAF-seq) and bulked segregation analysis (BSA). To facilitate this, we conducted a comparative analysis of two wheat germplasms, Pubing3228 and Jing4839, known to exhibit marked discrepancies in spike shape. By leveraging this methodology, we successfully procured 2,810,474 SLAF tags, subsequently resulting in the identification of 187,489 single nucleotide polymorphisms (SNPs) between the parental strains. We subsequently employed the SNP-index association algorithm alongside the extended distribution (ED) association algorithm to detect regions associated with the trait. The former algorithm identified 24 trait-associated regions, whereas the latter yielded 70. Remarkably, the intersection of these two algorithms led to the identification of 25 trait-associated regions. Amongst these regions, we identified 399 annotated genes, including three genes harboring non-synonymous mutant SNP loci. Notably, the APETALA2 (AP2) transcription factor families, which exhibited a strong correlation with spike type, were also annotated. Given these findings, it is plausible to hypothesize that these genes play a critical role in determining spike shape. In summation, our study contributes significant insights into the genetic foundation of grain number per spike. The molecular markers and candidate regions we have identified can be readily employed for marker-assisted breeding endeavors, ultimately leading to the development of novel wheat cultivars possessing enhanced yield potential. Furthermore, conducting further functional analyses on the identified genes will undoubtedly facilitate a comprehensive elucidation of the underlying mechanisms governing spike development in wheat.

## 1 Introduction

Wheat (*Triticum aestivum* L.), a globally cultivated staple crop, serves as a primary food source for approximately 35% of the world’s population. Given its status as the most abundant and traded food commodity, its economic significance cannot be understated (Guo et al., 2018). The spike, an essential reproductive structure of wheat, assumes a critical role in grain production and maintenance (Faris et al., 2014). Previous investigations have extensively explored the association between spike morphology and seed yield, specifically focusing on spike densities (SD), spike length (SL), and spikelet number per spike (SNS) (Chai et al., 2019; Li T. et al., 2021; You et al., 2021; Yu et al., 2022). The shape of the wheat spike exhibits a direct correlation with yield and is thus considered a pivotal agronomic trait during the process of domestication and selective breeding of wheat. Notably, the characteristics of the spike, including grain number per spike, exert a direct influence on yield levels. Therefore, gaining a comprehensive understanding of the genetic regulation underlying grain number per spike in wheat holds immense theoretical significance and practical value (Cui et al., 2012).

A comprehensive understanding of the physiological, genetic, and developmental mechanisms governing spikelet morphology is of paramount importance, as it not only contributes to the augmentation of spikelet numbers but also facilitates the improvement of spikelet fruiting or setting rates (Slafer et al., 2015; Gámez et al., 2020). However, conventional breeding techniques employed to enhance wheat yields encounter significant challenges, given the considerable costs and labor-intensive nature associated with incorporating these traits into extensive germplasm collections (Cui et al., 2012). Consequently, there arises a pressing need to explore innovative methodologies that can effectively elevate wheat yield potential.

The utilization of molecular markers plays a pivotal role in molecularly assisted selection (MAS) for plant breeding, as it establishes a link between phenotypes and molecular markers. Over time, various types of molecular markers have been developed, encompassing the first-generation markers such as restriction fragment length polymorphism (RFLP), random amplified polymorphic DNA (RAPD), and amplified fragment length polymorphism (AFLP), as well as the second-generation markers like simple sequence repeats (SSR) and inter-simple sequence repeats (ISSR). Nonetheless, these markers exhibit limitations in terms of throughput, accuracy, time consumption, labor intensiveness, and cost (Zheng et al., 2018; Clifton-brown et al., 2019; Chen et al., 2022). To surmount these challenges, a third generation of molecular markers known as SNPs has emerged. SNPs are DNA sequence variants that arise from a single nucleotide alteration in the genome sequence. They are typically distributed extensively across the genome and represent the most abundant form of genomic polymorphism, thereby making SNP markers the densest available marker type. The utilization of high-throughput DNA sequencing technologies enables the rapid and efficient identification of a vast number of SNPs in species, facilitating genotyping at high marker densities (Graner et al., 2013). Moreover, SNPs are often employed for population structure determination and linkage disequilibrium (LD) analysis (Jain et al., 2014). Concurrently, insertion/deletion (InDel) markers, which are also employed for fine mapping and marker-assisted selection in diverse crops (Liang et al., 2011; Wang et al., 2012; Das et al., 2015), can exert an influence on gene expression and function, given their location within coding and regulatory regions (Jain et al., 2014). Variants present within these regions can induce alterations in protein function and modulate gene expression, as exemplified by SNPs located on exons of the *GA2ox* gene, impacting spikelet sterility in wheat (Alqudah et al., 2020). Consequently, the discovery of SNPs assumes paramount significance for investigating genomic variation in crop species (Shen et al., 2017). In a genome-wide association study conducted on wheat, ten stable SNPs were identified, demonstrating associations with seeding emergence rate (SER) and tiller number at various fertility stages. Ultimately, a subset of these alleles exhibited efficacy in enhancing the seeding emergence rate and tiller number at diverse fertility stages (Chen et al., 2017).

SNPs have emerged as a highly effective tool for plant breeding through the implementation of MAS. However, the application of SNP-based array technology presents certain limitations in the detection of novel loci. To address this, the development of SLAF-seq markers has provided a promising solution, offering advantages such as deep sequencing, reduced sequencing costs, optimized marker efficiency, and suitability for large populations (Sun et al., 2013; Wang et al., 2022). In conjunction with BSA techniques, the SLAF-BSA method has been successfully employed for the identification of major QTLs associated with specific traits in various plant species. Unlike individual trait analysis, the BSA technique circumvents this requirement by focusing on individuals exhibiting contrasting extreme phenotypes. This integrated approach offers a cost-effective and accurate means to elucidate the genetic architecture underlying target traits in plant species lacking a reference genome (Michenlmore and Kesseli, 1991; Du et al., 2019). The efficacy of the SLAF-BSA method has been demonstrated across diverse plant species, including peas, oilseed rape, and pepper, where it has successfully identified QTL profiles and candidate genes associated with specific traits such as leaf shape, seed weight, first flowering node, and resistance to blast root rot. Thus, the SLAF-BSA method emerges as a potent tool for the discovery of genetic markers linked to desirable traits, ultimately facilitating plant breeding endeavors (Geng et al., 2016; Xu et al., 2016; Zhang et al., 2018; Zheng et al., 2018).

Pubing3228, a novel wheat germplasm derived from the hybridization of common wheat and *Agropyron*, exhibits numerous superior agronomic traits, particularly in relation to spike characteristics. However, the molecular basis governing the spike traits of Pubing3228 remains largely unexplored. Therefore, the primary objective of this investigation was to establish a high-resolution genetic map utilizing the SLAF-BSA approach in two wheat germplasms, namely Pubing3228 and Jing4839, in addition to F_2_ population generated from two exceptional wheat cultivars. By analyzing the extreme spike types observed in the two wheat germplasms, we aimed to identify stable molecular markers and candidate regions with the potential to contribute to future endeavors in fine mapping and gene cloning, specifically targeting key genes associated with enhanced wheat yield. This study represents a significant advancement in our comprehension of the genetic mechanisms underlying wheat yield traits, thereby paving the way for the development of more efficient strategies for wheat breeding and cultivation.

## 2 Materials and methods

### 2.1 Plant material and phenotypic data collection

**Figure 1:**
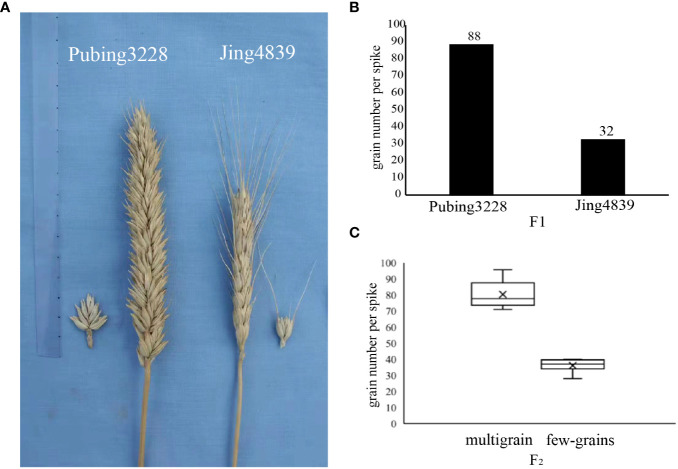
Wheat parental and hybrid second-generation spike counts. **(A)** Morphological characteristics of two varieties of paternal and maternal parent. **(B)** Average grain number of two paternal and maternal parent wheat varieties. **(C)** The number of two extreme characteristics after F_2_ generation trait separation.

Pubing3228, a genetically stable wheat derivative resulting from the cross between wheat and *A. cristatum*, exhibits desirable spike traits such as a long spike, high number of spikelets per spike, and high grain number per spike. This particular wheat germplasm was developed by the research team led by Lihui Li at the Chinese Academy of Agricultural Science. In order to explore the phenotypic differences in spike traits, two wheat germplasms, Pubing3228 and Jing4839, were carefully selected for a cross. The resulting second-generation offspring served as the sequencing material for the present study. In a field environment, the materials were planted in randomized complete blocks with three replications. Each block consisted of three rows, with each row measuring 2 meters in length and maintaining a 30 cm distance between rows. Standard local practices were followed for all field management activities. At the stage of physiological maturity, 10 representative plants per genotype from each replication were harvested and manually threshed. The grain number per spike (GNS) was subsequently recorded. The distinctive characteristics of the two wheat spike types are visually presented in **Figure 1**.

### 2.2 SLAF library construction and high-throughput sequencing

#### 2.2.1 Enzyme digestion protocol design

To select the most suitable enzyme sectioning scheme, the researchers utilized enzyme section prediction software to predict the enzyme sections of the reference genome. The selection principles were based on four criteria. Firstly, the proportion of enzyme sections located in repetitive sequences was kept as low as possible. Secondly, the enzyme sections were distributed as evenly as possible on the genome. Thirdly, the length of the enzyme sections matched the specific experimental system (Davey et al., 2013). Finally, the number of enzyme sections (SLAF tags) obtained was expected to meet the target number of tags.

#### 2.2.2 Experimental procedure

The genomic DNA of each qualified sample was digested using the optimal digestion protocol that was selected through the enzyme section prediction software. The resulting enzymatic fragments, or SLAF tags, were subjected to several steps including 3′-end addition A treatment, ligation of Dual-index sequencing connectors, PCR amplification, purification, mixing, gum cutting to select the target fragments, and library quality control. Finally, the libraries were sequenced using Illumina HiSeqTM with PE125bp sequencing.

#### 2.2.3 SLAF-BSA

For the BSA analysis, the spikes of both parental lines and selected spikes from the F_2_ population were segregated into two distinct pools, namely pools aa and ab. The identification of candidate genes was carried out utilizing two algorithms: the SNP-index association algorithm and the ED association algorithm. The SNP-index method, which is a recently published approach for marker association analysis, relies on the detection of genotype frequency disparities between the pools (Fekih et al., 2013; Takagi et al., 2013). The primary aim of this method is to discern significant deviations in genotype frequencies within mixed pools by employing the ΔSNP-index statistic. The calculation procedure can be summarized as follows:


SNPindex(aa)=Maa/(Maa+Paa)

Maa indicates that the aa pool is derived from the depth of the female parent; Paa indicates that the aa pool is derived from the depth of the male parent.


SNPindex(ab)=Mab/(Mab+Pab)

Mab indicates that the ab pool is derived from the depth of the female parent; Pab indicates that the ab pool is derived from the depth of the male parent.


ΔSNPindex=SNPindex(aa)−SNPindex(ab)

In this investigation, the BSA technique was employed to ascertain candidate genes associated with wheat yield. To facilitate this analysis, wheat spikes obtained from both parental lines and selected spikes from the F_2_ population were carefully segregated into two distinct pools denoted as pools aa and ab. Subsequently, two algorithms, namely SNP-index and ED, were utilized for candidate gene selection. The SNP-index algorithm, a recent development in marker association analysis, exploits the disparities in genotype frequencies observed between the mixed pools, quantified by the ΔSNP-index statistic. Conversely, the ED algorithm employs sequencing data to identify markers that exhibit significant differences between the pools, thereby facilitating the assessment of the genomic region’s association with the trait of interest. It is important to note that the ED value for non-target loci is expected to be 0, given that the BSA project constructs two hybrid pools that are primarily identical except for the variations present in the target trait-related loci. A higher ED value indicates a more pronounced distinction between the two hybrid pools, thereby signifying a stronger association with the trait under investigation.


$$ED=\sqrt{{\left(A_{mut}-A_{wt}\right)}^{2}+{\left(C_{mut}-C_{wt}\right)}^{2}+{\left(G_{mut}-G_{wt}\right)}^{2}+{\left(T_{mut}-T_{wt}\right)}^{2}}$$

Amut represents the frequency of A bases observed in the mutant mixing pool, while Awt signifies the frequency of A bases in the wild-type mixing pool. Similarly, Cmut denotes the frequency of C bases in the mutant mixing pool, whereas Cwt corresponds to the frequency of C bases in the wild-type mixing pool. Furthermore, Gmut indicates the frequency of G bases in the mutant mixing pool, while Gwt represents the frequency of G bases in the wild-type mixing pool. Lastly, Tmut denotes the frequency of T bases observed in the mutant mixing pool, and Twt signifies the frequency of T bases in the wild-type mixing pool.

## 3 Result

Significant distinctions were observed between Pubing3228 and Jing4839 in terms of spike length, morphology per spikelet, awning length, and number of grains per spike, as depicted in **Figures 1A, B**. Pubing3228 exhibited longer spikes, larger morphology per spikelet, and an absence of awning throughout the spike, whereas Jing4839 displayed smaller spikes, smaller morphology per spikelet, and longer awning throughout the spike. The average grain count per spike was determined to be 88 for Pubing3228 and 32 for Jing4839.

In order to identify potential molecular markers and candidate regions associated with the target trait, a mixed pool sample was created by crossing the two wheat varieties, and the resulting F_2_generation was classified into two distinct types based on grain number. The multi-spike grain type exhibited an average of 80 grains per spike, whereas the few-spike grain type had an average of 36 grains per spike, as illustrated in **Figure 1C**.The statistical information regarding the seed grain situation of the F_2_ generation is presented in **Supplementary Table 1**. Subsequently, the second generation progeny was subjected to sequencing and subsequent analysis to construct the mixed pool sample.

### 3.1 Characterization of SLAF sequencing data and SNPs

**Figure 2:**
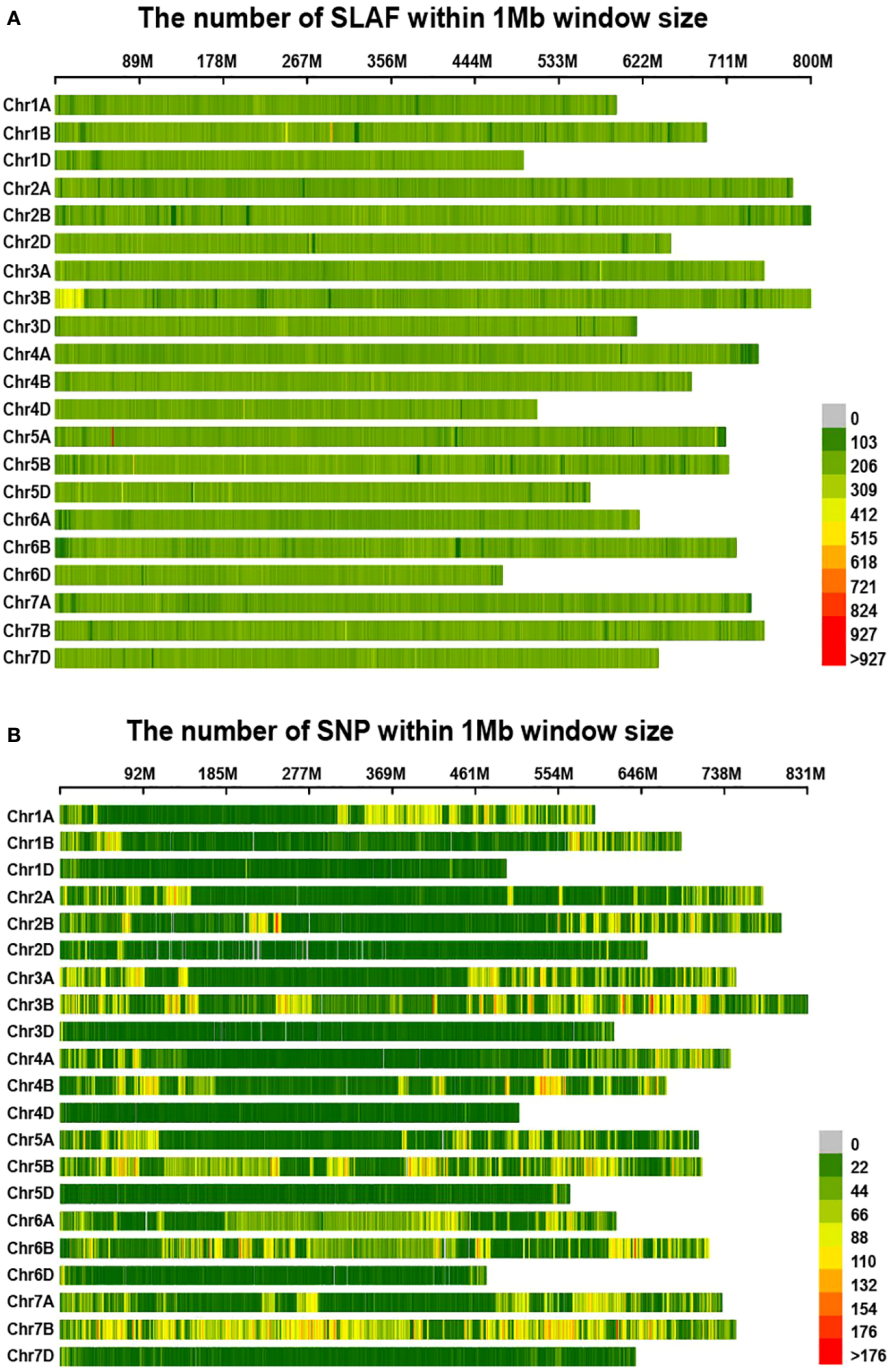
Distribution of SLAF-tags **(A)** and SNPs **(B)** on each reference chromosome in wheat. **(A, B)** The horizontal axis of the figure illustrates the chromosomal lengths, where each yellow band represents an individual chromosome. The genome is partitioned into 1 Mb segments. The shading intensity of each window corresponds to the number of labels contained within it, with darker windows indicating a higher label count, while lighter windows indicate a lower label count.

In this investigation, the RsaI enzyme was employed to enzymatically fragment the reference genome, targeting specific sections with a length range of 464-484 base pairs. This enzymatic cleavage procedure yielded a total of 311,781 SLAF tags. Subsequently, the parental and mixed pool samples underwent the SLAF-seq sequencing protocol, resulting in the generation of 239.26 million reads after applying a quality filtering criterion of 80% Q30. Among these reads, 2,810,474 SLAF tags were successfully identified, exhibiting a sequencing depth of 11.67X and 8.32X for each parental sample, and 20.10X and 24.15X for the mixed pool samples, respectively. Notably, these SLAF tags were evenly distributed across the 21 chromosome pairs, as illustrated in **Figure 2A**. Furthermore, a total of 187,489 SNPs were detected between the parental samples, while 164,018 SNPs were identified within the mixed pool samples, as depicted in **Figure 2B**. A comprehensive summary of these findings is presented in **Table 1**.

**Table 1:** SLAF tag statistics.

ID	SLAF number	Total depth	Average depth
M	1,463,703	17,074,359	11.665,2
P	1,519,806	12,647,895	8.322,0
aa	1,851,584	37,225,010	20.104,4
ab	1,936,123	46,749,748	24.146,1
Total	2,810,474	113,697,012	40.454,7

The table displays the following information: ID (the unique identifier of the sample), SLAF number (the number of SLAF tags in the corresponding sample), Total depth (the total sequencing depth of the SLAF tags in the corresponding sample, i.e., the total number of reads developed from the SLAF tags), and Average depth (the average number of sequencing reads of the corresponding sample on each SLAF).

### 3.2 High-quality SNP screening

Prior to conducting the association analysis, a comprehensive screening of SNP loci was carried out. A total of 88,211 high-quality SNP loci were identified by applying filters to exclude loci with multiple genotypes, loci with less than 4 read support, loci with consistent genotypes among mixed pools, and loci with recessive mixed pool genes not inherited from the recessive parent (**Table 2**).

**Table 2:** SNP filtering statistics.

Total_SNP	MAL	LRS	GCLMP	UPFL	HSNP
361,030	643	167,992	12,596	91,588	88,211

MAL, Multiple allele loci; LRS, Loci with reading support less than 4; GCLMP, Genotypically congruent loci in mixed pools; UPFL, Use of parentally filtered loci; HSNP, High-quality SNPs.

### 3.3 Correlation analysis

The ED algorithm, a widely utilized approach, is employed to detect markers that exhibit significant differences between pools and to evaluate regions associated with specific traits (Hill et al., 2013). In the context of the BSA project, two mixed pools are generated to identify disparities in loci related to the target trait, while minimizing variations in non-target loci. Consequently, the ED value for non-target loci is expected to converge towards 0. To mitigate background noise, the fourth power of the initial ED was utilized as the associated value, and the SNPNUM method was employed to fit the ED values. The association threshold for analysis was determined as 0.07, calculated as the median plus three standard deviations (SD) of the fitted values for all loci (Hill et al., 2013).

**Figure 3:**
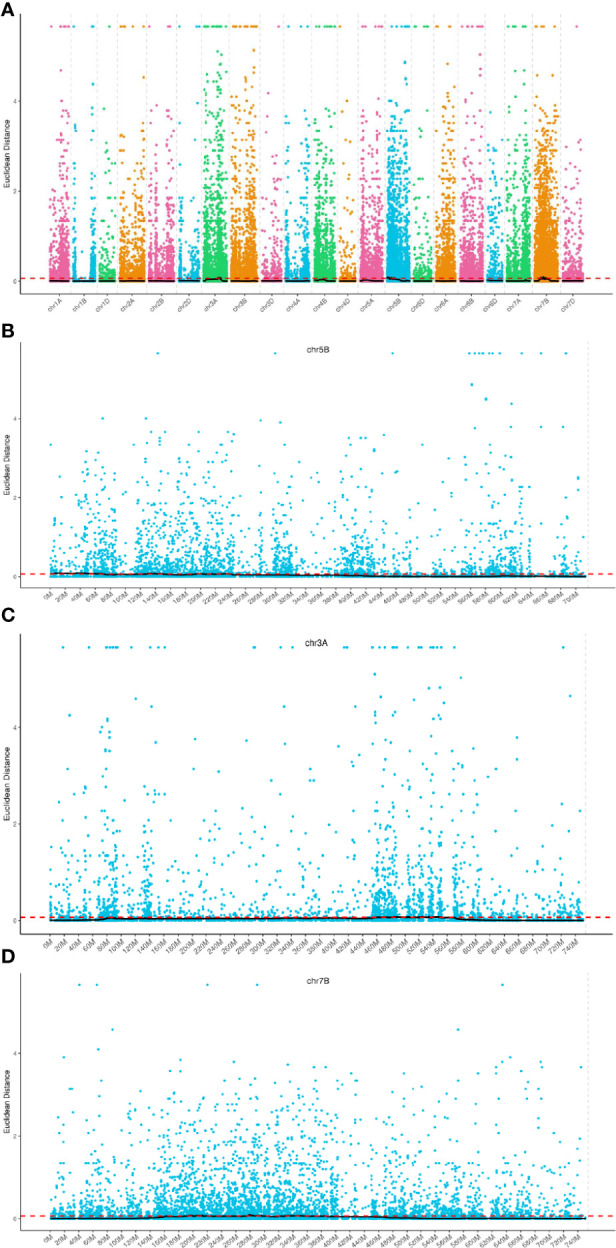
Distribution of ED association values on chromosomes. **(A–D)** The horizontal axis represents the names of the chromosomes. Each colored dot represents the ED value of a specific SNP locus, while the black line corresponds to the fitted ED value. The red dashed line indicates the threshold for significant association. A higher ED value indicates a stronger association at that particular point.

Seventy regions encompassing a total of 1,850 genes were identified, with these regions spanning a cumulative length of 241.70 Mb (**Figure 3A**). Among these genes, 10 harbored non-synonymous mutant SNP loci. Chromosome-wise distribution revealed that 647 genes were located on chromosome 3A, 913 genes on chromosome 5B, and 290 genes on chromosome 7B. For further analysis, detailed information on the screened genes is provided in **Supplementary Table 2** (**Figures 3B–D**).

### 3.4 SNP-index method association results

#### 3.4.1 SNP index analysis

**Figure 4:**
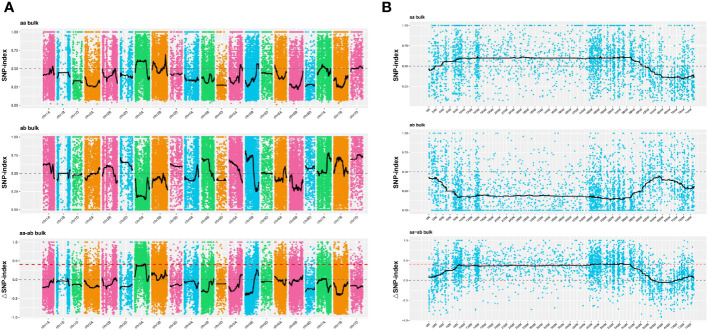
Distribution of SNP-index association values on chromosomes. **(A, B)** The x-axis corresponds to the chromosome names, while the y-axis represents the calculated ΔSNP-index values. The colored dots on the graph represent the calculated ΔSNP-index values, while the black lines indicate the fitted ΔSNP-index values. The top panel displays the distribution of SNP-index values specifically for the recessive mixed pool, whereas the middle panel depicts the distribution of SNP-index values for the dominant mixed pool. Lastly, the bottom panel illustrates the distribution of ΔSNP-index values, with the red line indicating the threshold line set at the 99th percentile.

The SNP index method, a recent development in marker association analysis based on genotype frequency differences between mixed pools, was utilized in this study (Fekih et al., 2013; Takagi et al., 2013). To mitigate false positive findings, the ΔSNP-index statistic was employed, and fitting of ΔSNP-index values for markers located on the same chromosome was conducted using their genomic positions. The SNPNUM method was employed to fit the ΔSNP-index values, and regions surpassing the association threshold were considered as trait-associated regions. **Figure 4** depicts the distribution of SNP-index and ΔSNP-index for each of the two mixed pools. By employing the 99th percentile of the fitted ΔSNP-index (0.40), a total of 24 regions spanning 44.33 Mb and encompassing 400 genes were identified (**Supplementary Table 3**).

### 3.5 Candidate area screening

#### 3.5.1 SNP annotation of candidate regions

A comparison was conducted between the association results obtained from the ED method and the SNP-index method, and the intersecting genes were subjected to annotation. Within this intersection, three SNPs were identified, exhibiting non-synonymous mutations between the parental lines, thus directly linking them to the target traits (**Supplementary Table 4**). These three genes were identified as *TraesCS3A01G260600, TraesCS3A01G261000*, and *TraesCS3A01G310500*, all of which exhibited up-regulation in wheat.

### 3.6 Functional notes on candidate areas

**Figure 5:**
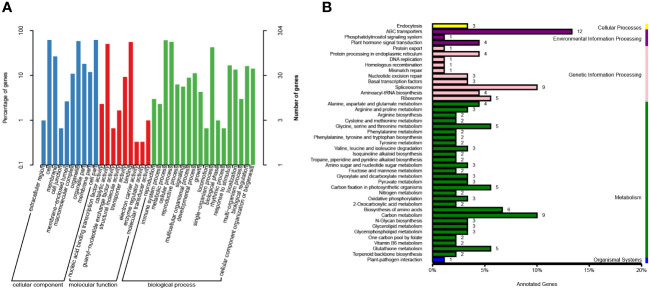
Gene KEGG and GO annotations in candidate regions. **(A)** Gene annotations based on the GO database. The proportion of genes belonging to the three main categories of GO classification is represented on the left Y-axis, while the number of genes in each category is depicted on the right Y-axis. **(B)** Gene annotations derived from the KEGG database. The Y-axis represents the top 50 metabolic pathways, whereas the X-axis displays the number of genes annotated to these pathways and their ratio in relation to the total number of annotated genes.

The candidate genes within the identified intervals underwent comprehensive annotation using various databases such as Gene Ontology (GO, https://geneontology.org/), Kyoto Encyclopedia of Genes and Genomes (KEGG, https://www.genome.jp/kegg/), and others, facilitated by the BLAST software. This thorough annotation allowed for efficient screening of candidate genes, resulting in the annotation of 399 genes within the candidate region (**Figure 5**, Please refer to **Supplementary Table 5** for additional details). **Supplementary Table 5** presents the annotated genes that exhibited non-synonymous mutant SNPloci. Among them, three genes were annotated in theNon-Redundant Protein Database (NR, https://www.ncbi.nlm.nih.gov/refseq/about/nonredundantproteins/) andTranslation of EMBL (trEMBL, https://www.ebi.ac.uk/Tools/st/) databases, while no annotation was found in the KEGG database. *TraesCS3A01G310500* was annotated as a B3 domain-containing protein in the SwissProt database (https://ngdc.cncb.ac.cn/databasecommons/database/id/5614), and *TraesCS3A01G260600.1* was annotated as being involved in lipid transport and metabolism, according to the Clusters of Orthologous Groups of proteins (COG, https://www.ncbi.nlm.nih.gov/research/cog/) database.

GO annotation was performed for a total of 399 genes located within the candidate region. These genes were annotated based on their classification into cellular component (CC), biological process (BP), and molecular function (MF) categories. Among the CC annotations, the most frequently assigned positions included cell (188), cell part (188), organelle (177), intracellular organelle (177), cytoplasm (169), membrane (86), and others. In terms of BP, the commonly annotated processes were metabolic process (185), cellular process (175), and single-organism process (133). Regarding MF annotations, the predominant functional categories were binding (169), catalytic activity (153), and transferase activity (75) (**Supplementary Table 6**).

Furthermore, GO enrichment analysis was performed on the 10 genes with the smallest KS (Kolmogorov-Smirnov) values in CC, BP, and MF. These genes exhibited enrichment in specific GO terms. In CC, the enriched terms included intracellular organelles (177), nucleus (56), cytoplasmic membrane-bounded vesicles (32), interchromatin granules (3), perinuclear region of cytoplasm (2), and others. In BP, the enriched terms comprised oxidation-reduction processes (14), negative regulation of DNA-templated transcription (4), recognition of pollen (3), arginyl-tRNAaminoacylation (2), and others. In MF, the enriched terms were primarily related to pyridoxal phosphate binding (8), diacylglycerol O-acyltransferase activity (3), arginine-tRNA ligase activity (2), and others. These genes were primarily associated with the activation of specific enzymatic activities, redox processes, and energy transfer. The nucleus and cytoplasm were identified as the main sites of action for these genes.

Genes in an organism collectively contribute to the execution of various biological functions, while pathways represent a coordinated sequence of interactions involving multiple genes. In order to gain insights into the functional characteristics of the 399 genes within the candidate regions, KEGG annotation was conducted (**Supplementary Table 7**). The analysis revealed that the majority of genes (191) were annotated within the categories of Environmental Information Processing, Genetic Information Processing, and Metabolism. Within the Environmental Information Processing category, 12 genes were assigned to the ABC transporters pathway. In the Genetic Information Processing category, 9 genes were associated with the Spliceosome pathway, and 5 genes were linked to the Ribosome pathway. In the Metabolism category, 9 genes were annotated in the Carbon metabolism pathway, 6 genes were involved in the Biosynthesis of amino acids pathway, and 4 genes were associated with each of the following pathways: Glycine, serine and threonine metabolism; Carbon fixation in photosynthetic organisms; and Glutathione metabolism.

## 4 Discussion

In wheat breeding programs, the identification of genes or QTL associated with yield-related traits is of great importance, as these traits often exhibit higher heritability compared to yield itself. This genetic information is crucial for understanding the underlying mechanisms of yield and facilitating the genetic improvement of wheat varieties (Li et al., 2022). Among the yield-related traits, spike-related traits hold promise for enhancing seed yield. However, conventional screening of these traits in large isolated populations can be laborious, costly, and inefficient. As a result, the adoption of molecular-assisted selection (MAS) or genetic selection has gained popularity as an efficient breeding approach. A comprehensive understanding of the genetic basis underlying yield traits, including wheat spike morphology, is essential for enhancing wheat yield. In this regard, the utilization of genetic resources such as wheat genome sequences and SNP platforms for wheat breeding plays a pivotal role in the development of improved wheat varieties (Malik et al., 2021).

SLAF-seq, a cost-effective technique, has proven to be highly valuable in generating a large number of polymorphic markers, thereby enabling the construction of high-density genetic maps for plant species with extensive genomes (Sun et al., 2013). Compared to traditional PCR-based methods, numerous studies have demonstrated that SLAF-seq significantly enhances the accuracy and efficiency of QTL mapping (Zheng et al., 2018). To improve mapping precision and narrow down candidate regions, BSA has been employed, which involves the selection of individuals with extreme phenotypes. BSA has been successfully utilized as a molecular marker in various organisms, and protocols have been developed specifically in model plants such as Arabidopsis and rice (Song et al., 2017). Classical BSA analysis reduces the cost of second-generation sequencing by pooling equimolar amounts of DNA from individuals exhibiting the same trait (Zhu et al., 2021). In our study, we performed SLAF-seq and obtained a total of 2,810,474 SLAF tags, resulting in the identification of 187,489 SNPs between the parental lines and 164,018 SNPs between the mixed pools. Using the SNP-index association algorithm, we identified 24 trait-associated marker regions spanning a cumulative length of 44.33 Mb. Additionally, employing the ED association algorithm led to the identification of 70 trait-associated regions. Within these association regions, a total of 399 genes were annotated. Notably, three genes harbored non-synonymous mutant SNP loci, suggesting a direct association with the trait under investigation. These genes were considered as promising candidates for subsequent analyses.

Further analysis of the 399 annotated genes revealed that the KEGG enrichment results highlighted the enrichment of ABC transporters, which are responsible for facilitating the transportation of various substances across membranes. Among these transporters, certain inward transporters play a vital role in the transport of nutrients, including amino acids and sugars, from the extracellular environment to the intracellular matrix. This process promotes cell growth and viability (Theodoulou and Kerr, 2015). Additionally, several amino acid metabolite pathways, such as Glycine, serine, and threonine metabolism, Alanine, aspartate, glutamate metabolism, Arginine and proline metabolism, and Arginine biosynthesis, were identified through KEGG enrichment analysis. Furthermore, specific metabolites associated with cell wall, respiratory, and protective functions were found to be correlated with genotypic superiority and yield stability (Vergara-Diaz et al., 2020).

Environmental factors, including temperature, photoperiod, water, and mineral availability, have been shown to regulate spikelet differentiation rate, apical spikelet formation, and ultimately determine the final grain number. Carbon fixation and metabolic pathways were also enriched in our analysis. Gámez et al. (2020) demonstrated that a significant portion of the carbon accumulated in wheat seeds is derived from the spikelet. They further found that the spikelet exhibits twice the capacity of the flag leaf lamina in re-fixing respired CO_2_ and shows higher rates of gross photosynthesis and respiration compared to the flag leaf.

The GO analysis revealed the enrichment of terms related to hormones, kinases, and floral development, particularly in response to growth hormone (GO:0060416). This finding underscores the role of growth hormone, a key plant hormone, in promoting cell polarity establishment, cell elongation, and influencing spike type and the number of grains per spike (Gallavotti et al., 2008). Moreover, auxin transport proteins were identified as critical for auxin distribution during wheat spike development, suggesting their significance in regulating this process (Li et al., 2018).

The inflorescence branching pattern in wheat spikes is a crucial trait that significantly impacts floret formation and overall productivity. Transcription factor annotation of genes within candidate regions has revealed a noteworthy similarity to members of the APETALA2 (AP2)-like transcription factor family, particularly the gene *TraesCS3A01G259900*, which bears a close relationship to the Q gene (Simons et al., 2006). The Q gene is considered a major gene in wheat domestication, and its q allele is prevalent in cultivated wheat varieties. Extensive research has highlighted the q allele’s influence on various traits, including subcompact spike phenotype, rachis fragility, gum toughness, plant height, and flowering time. Moreover, it is associated with deltoid spike morphology and non-free-threshing grains (Faris et al., 2005).Loss-of-function alleles at the q locus in wheat exhibit an extended developmental stage of the rachilla, leading to a significant increase in the number of florets per spikelet. MicroRNA172 (miRNA172) interactions with its target genes, particularly the genes encoding AP2-like transcription factors such as the Q gene, play a pivotal role in cereal inflorescence development and structure (Debernardi et al., 2017; Greenwood et al., 2017). The AP2 transcription factor family, including the Q gene (*AP2L5*) and its associated paralog *AP2L2*, is regulated by miRNA172 and demonstrates critical and redundant functions in specifying axillary floral meristems (Debernardi et al., 2020). The proper balance of AP2-like gene expression, located on chromosomes 5AL (Q), 2AL (*AP2L2-2A*), 2BL (*AP2L2-2B*), and 2DL (*AP2L2*), is essential for spikelet and floret development (Adonina et al., 2021). Structural reorganization of a chromosome during hybridization can disrupt this balance, thereby offering an opportunity to modify spikelet structure and potentially improve grain yield by manipulating this regulatory module.The*WFZP* gene, a member of the *AP2/ERF* transcription factor family, has been experimentally demonstrated to regulate spikelet development and exhibits conserved functionality between wheat and Brachypodium. Mutations in *WFZP* can affect spikelet development by activating *TaGW5* or directly or indirectly inhibiting *TaGW8*, thereby influencing grain width and weight. Enhancing wheat grain yield remains a challenging task in breeding programs, and the identification of valuable genes and favorable alleles through screening represents a crucial approach towards achieving this goal (Li T. et al., 2021).

## Data availability statement

The original contributions presented in the study are publicly available. This data can be found here: National Genomics Data Center, accession number CRA015054.

## Author contributions

JW: Conceptualization, Formal analysis, Funding acquisition, Investigation, Software, Supervision, Validation, Writing – original draft. EW: Data curation, Investigation, Writing – review & editing. SC: Data curation, Validation, Writing – review & editing. AM: Investigation, Validation, Visualization, Writing – review & editing.
